# 2,2′-Bipyrimidine-1,1′-diium bis­(tri­iodide)–2,2′-bipyrimidine–water (1/2/2)

**DOI:** 10.1107/S1600536811034982

**Published:** 2011-09-03

**Authors:** Kwang Ha

**Affiliations:** a School of Applied Chemical Engineering, The Research Institute of Catalysis, Chonnam National University, Gwangju 500-757, Republic of Korea

## Abstract

In the crystal of the title compound, C_8_H_8_N_4_^2+^·2I_3_^−^·2C_8_H_6_N_4_·2H_2_O, inversion centres are located at the centroids of the central C—C bonds of the cation and the bpym mol­ecules, as well as at the central I atoms of both anions. Inter­molecular O—H⋯N and N—H⋯O hydrogen bonds are observed in the crystal structure.

## Related literature

For related structures, see: Fialho De Assis *et al.* (1996 ▶); Kochel (2005 ▶). For the synthesis and crystal structure of [Mn(C_8_H_6_N_4_)_3_](I_3_)_2_·CH_3_NO_2_, see: Ha (2011 ▶).
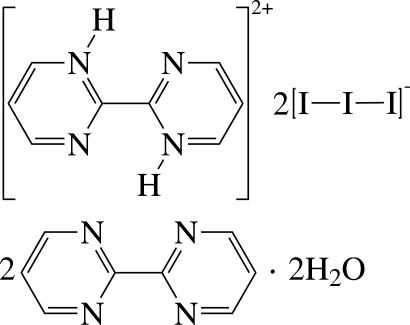


## Experimental

### 

#### Crystal data

C_8_H_8_N_4_^2+^·2I_3_^−^·2C_8_H_6_N_4_·2H_2_O
*M* *_r_* = 1273.95
Triclinic, 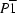

*a* = 8.964 (2) Å
*b* = 9.013 (2) Å
*c* = 12.112 (3) Å
α = 77.265 (5)°
β = 76.881 (5)°
γ = 76.287 (4)°
*V* = 911.3 (4) Å^3^
*Z* = 1
Mo *K*α radiation
μ = 5.15 mm^−1^
*T* = 200 K
0.35 × 0.21 × 0.16 mm

#### Data collection

Bruker SMART 1000 CCD diffractometer
Absorption correction: multi-scan (*SADABS*; Bruker, 2000 ▶) *T*_min_ = 0.769, *T*_max_ = 1.000
5688 measured reflections
3505 independent reflections
2993 reflections with *I* > 2σ(*I*)
*R*_int_ = 0.018

#### Refinement

*R*[*F* ^2^ > 2σ(*F* ^2^)] = 0.053
*wR*(*F* ^2^) = 0.132
*S* = 1.31
3505 reflections
202 parameters
H-atom parameters constrained
Δρ_max_ = 1.29 e Å^−3^
Δρ_min_ = −1.67 e Å^−3^

### 

Data collection: *SMART* (Bruker, 2000 ▶); cell refinement: *SAINT* (Bruker, 2000 ▶); data reduction: *SAINT*; program(s) used to solve structure: *SHELXS97* (Sheldrick, 2008 ▶); program(s) used to refine structure: *SHELXL97* (Sheldrick, 2008 ▶); molecular graphics: *ORTEP-3* (Farrugia, 1997 ▶) and *PLATON* (Spek, 2009 ▶); software used to prepare material for publication: *SHELXL97*.

## Supplementary Material

Crystal structure: contains datablock(s) global, I. DOI: 10.1107/S1600536811034982/im2310sup1.cif

Structure factors: contains datablock(s) I. DOI: 10.1107/S1600536811034982/im2310Isup2.hkl

Additional supplementary materials:  crystallographic information; 3D view; checkCIF report

## Figures and Tables

**Table 1:** Hydrogen-bond geometry (Å, °)

*D*—H⋯*A*	*D*—H	H⋯*A*	*D*⋯*A*	*D*—H⋯*A*
O1—H1*A*⋯N3^i^	0.84	1.88	2.721 (11)	178
O1—H1*B*⋯N2^ii^	0.84	2.09	2.837 (10)	149
N1—H1*N*⋯O1^iii^	0.88	2.59	3.450 (11)	166

Symmetry codes: (i) 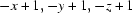; (ii) 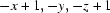; (iii) 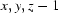.

**Fig. 1.**
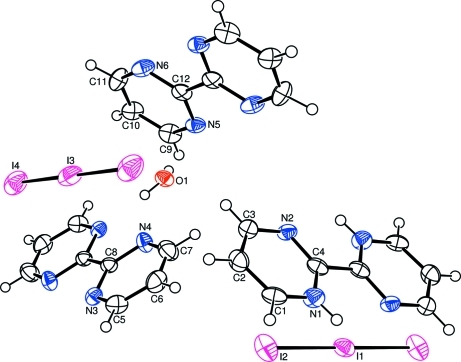
Molecular structure of the title compound with displacement ellipsoids drawn at the 50% probability level. H atoms are shown as small circles of arbitrary radius. Unlabelled atoms are generated by the application of the inversion centres.

**Fig. 2.**
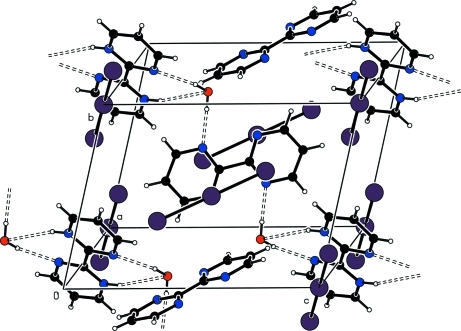
View of the unit-cell contents of the title compound. Hydrogen-bond interactions are drawn with dashed lines.

## supplementary crystallographic information

### Comment

The title compound, (C_8_H_8_N_4_)^2+^(I_3_^-^)_2_.2C_8_H_6_N_4_ × 2 H_2_O, consists of a diprotonated 2,2'-bipyrimidinium cation, two discrete I_3_^-^ anions, two 2,2'-bipyrimidine (bpym) and two water solvent molecules (Fig. 1). Single crystals of the compound were unexpectedly obtained as a byproduct from the reaction of MnI_2_ with 2,2'-bipyrimidine (Ha, 2011), and the structure is related to that of the compounds (C_8_H_8_N_4_)[ReCl_6_].2H_2_O (Kochel, 2005) and (C_10_H_9_N_2_)(I_3_) (Fialho De Assis *et al.*, 1996).

The asymmetric unit contains one half of the formula unit. Inversion centres are located at the centroids of the central carbon carbon bond of the cation and the bpym molecules. Therefore the two pyrimidine rings in each moiety are exactly parallel. The central I atoms (I1 and I3) of the anions also occupy crystallographic inversion centres: the anions are linear and the I—I distances are almost equal. Intermolecular O—H···N and N—H···O hydrogen bonds stabilize the crystal structure and the cation reveals short intramolecular N—H···N hydrogen bonds (Fig. 2 and Table 1). Moreover, there are weak intermolecular π-π interactions between pyrimidine rings, with a shortest ring centroid-centroid distance of 5.239 (6) Å.

### Experimental

The title compound was unexpectedly obtained as a byproduct from the reaction of MnI_2_ (0.3086 g, 1.000 mmol) with 2,2'-bipyrimidine (0.1588 g, 1.004 mmol) in acetone (30 ml). After stirring of the reaction mixture for 3 h at room temperature and addition of pentane (30 ml), the formed precipitate was separated by filtration, washed with EtOH and ether, to give a brown powder (0.3621 g) (Ha, 2011). Single crystals of the title compound suitable for X-ray analysis were obtained by slow evaporation from the brown filtrate.

### Refinement

H atoms at nitrogen were observed in the Fourier maps but were nevertheless as H atoms at carbon positioned geometrically and allowed to ride on their respective parent atoms [C—H = 0.95 Å (CH) or 0.88 Å (NH) and *U*_iso_(H) = 1.2*U*_eq_(C, N)]. The H atoms of the solvent water molecules were located from the difference Fourier map then allowed to ride on their parent O atoms in the final cycles of refinement with O—H = 0.84 Å and *U*_iso_(H) = 1.5 *U*_eq_(O). The highest peak (1.29 e Å^-3^) and the deepest hole (-1.67 e Å^-3^) in the difference Fourier map are located 1.38 Å and 0.93 Å from the atoms I2 and I1, respectively.

### Crystal data

**Table:**

C_8_H_8_N_4_^2+^·2I_3_^−^·2C_8_H_6_N_4_·2H_2_O	*Z* = 1
*M**_r_* = 1273.95	*F*(000) = 586
Triclinic, *P*1	*D*_x_ = 2.321 Mg m^−^^3^
Hall symbol: -P 1	Mo *K*α radiation, λ = 0.71073 Å
*a* = 8.964 (2) Å	Cell parameters from 3632 reflections
*b* = 9.013 (2) Å	θ = 2.4–25.9°
*c* = 12.112 (3) Å	µ = 5.15 mm^−^^1^
α = 77.265 (5)°	*T* = 200 K
β = 76.881 (5)°	Block, redbrown
γ = 76.287 (4)°	0.35 × 0.21 × 0.16 mm
*V* = 911.3 (4) Å^3^	

### Data collection

**Table:**

Bruker SMART 1000 CCD diffractometer	3505 independent reflections
Radiation source: fine-focus sealed tube	2993 reflections with *I* > 2σ(*I*)
graphite	*R*_int_ = 0.018
φ and ω scans	θ_max_ = 26.0°, θ_min_ = 2.4°
Absorption correction: multi-scan (*SADABS*; Bruker, 2000)	*h* = −10→11
*T*_min_ = 0.769, *T*_max_ = 1.000	*k* = −11→11
5688 measured reflections	*l* = −13→14

### Refinement

**Table:**

Refinement on *F*^2^	Primary atom site location: structure-invariant direct methods
Least-squares matrix: full	Secondary atom site location: difference Fourier map
*R*[*F*^2^ > 2σ(*F*^2^)] = 0.053	Hydrogen site location: inferred from neighbouring sites
*wR*(*F*^2^) = 0.132	H-atom parameters constrained
*S* = 1.31	*w* = 1/[σ^2^(*F*_o_^2^) + (0.0114*P*)^2^ + 14.2699*P*] where *P* = (*F*_o_^2^ + 2*F*_c_^2^)/3
3505 reflections	(Δ/σ)_max_ < 0.001
202 parameters	Δρ_max_ = 1.29 e Å^−^^3^
0 restraints	Δρ_min_ = −1.67 e Å^−^^3^

### Special details

**Table:**

Geometry. All e.s.d.'s (except the e.s.d. in the dihedral angle between two l.s. planes) are estimated using the full covariance matrix. The cell e.s.d.'s are taken into account individually in the estimation of e.s.d.'s in distances, angles and torsion angles; correlations between e.s.d.'s in cell parameters are only used when they are defined by crystal symmetry. An approximate (isotropic) treatment of cell e.s.d.'s is used for estimating e.s.d.'s involving l.s. planes.
Refinement. Refinement of *F*^2^ against ALL reflections. The weighted *R*-factor *wR* and goodness of fit *S* are based on *F*^2^, conventional *R*-factors *R* are based on *F*, with *F* set to zero for negative *F*^2^. The threshold expression of *F*^2^ > σ(*F*^2^) is used only for calculating *R*-factors(gt) *etc*. and is not relevant to the choice of reflections for refinement. *R*-factors based on *F*^2^ are statistically about twice as large as those based on *F*, and *R*- factors based on ALL data will be even larger.

### Fractional atomic coordinates and isotropic or equivalent isotropic displacement parameters (Å^2^)

**Table:**

	*x*	*y*	*z*	*U*_iso_*/*U*_eq_	
I1	1.0000	0.0000	0.0000	0.0408 (3)	
I2	0.74916 (11)	0.26620 (10)	0.03957 (9)	0.0590 (3)	
I3	0.0000	0.5000	0.5000	0.0465 (3)	
I4	0.11198 (10)	0.59729 (11)	0.67614 (7)	0.0543 (3)	
N1	0.3970 (10)	0.1722 (10)	−0.0778 (7)	0.0340 (19)	
H1N	0.4476	0.1641	−0.1482	0.041*	
N2	0.3607 (9)	0.0652 (9)	0.1209 (7)	0.0281 (18)	
N3	0.4427 (9)	0.6152 (9)	0.3680 (7)	0.0273 (17)	
N4	0.4734 (10)	0.3448 (10)	0.4394 (7)	0.0324 (19)	
N5	0.1907 (9)	0.0208 (9)	0.5125 (7)	0.0293 (18)	
N6	−0.0604 (10)	0.0811 (10)	0.6308 (7)	0.0327 (19)	
C1	0.2819 (12)	0.2904 (12)	−0.0578 (9)	0.033 (2)	
H1	0.2550	0.3689	−0.1207	0.040*	
C2	0.1989 (12)	0.3036 (12)	0.0521 (9)	0.035 (2)	
H2	0.1168	0.3895	0.0664	0.042*	
C3	0.2421 (12)	0.1847 (12)	0.1401 (9)	0.035 (2)	
H3	0.1859	0.1880	0.2165	0.042*	
C4	0.4339 (11)	0.0663 (11)	0.0113 (9)	0.028 (2)	
C5	0.4007 (12)	0.5968 (13)	0.2747 (8)	0.033 (2)	
H5	0.3729	0.6850	0.2184	0.039*	
C6	0.3968 (13)	0.4517 (14)	0.2580 (9)	0.041 (3)	
H6	0.3698	0.4370	0.1903	0.049*	
C7	0.4334 (14)	0.3302 (13)	0.3431 (10)	0.042 (3)	
H7	0.4303	0.2292	0.3335	0.051*	
C8	0.4744 (11)	0.4879 (11)	0.4473 (8)	0.026 (2)	
C9	0.2583 (12)	0.0653 (12)	0.5860 (9)	0.035 (2)	
H9	0.3679	0.0607	0.5701	0.042*	
C10	0.1691 (12)	0.1169 (12)	0.6831 (8)	0.033 (2)	
H10	0.2159	0.1437	0.7371	0.040*	
C11	0.0077 (13)	0.1291 (13)	0.7005 (8)	0.035 (2)	
H11	−0.0562	0.1729	0.7641	0.043*	
C12	0.0361 (12)	0.0280 (11)	0.5389 (8)	0.029 (2)	
O1	0.5739 (8)	0.0741 (8)	0.6568 (6)	0.0321 (16)	
H1A	0.5705	0.1700	0.6472	0.048*	
H1B	0.6249	0.0175	0.7061	0.048*	

### Atomic displacement parameters (Å^2^)

**Table:**

	*U*^11^	*U*^22^	*U*^33^	*U*^12^	*U*^13^	*U*^23^
I1	0.0539 (7)	0.0402 (6)	0.0341 (5)	−0.0204 (5)	−0.0139 (5)	−0.0004 (4)
I2	0.0621 (6)	0.0466 (5)	0.0748 (7)	−0.0098 (4)	−0.0217 (5)	−0.0159 (4)
I3	0.0351 (6)	0.0436 (6)	0.0406 (6)	0.0054 (4)	0.0029 (4)	0.0102 (5)
I4	0.0437 (5)	0.0571 (5)	0.0466 (5)	0.0078 (4)	−0.0030 (4)	−0.0018 (4)
N1	0.035 (5)	0.032 (5)	0.031 (5)	−0.003 (4)	−0.007 (4)	0.001 (4)
N2	0.029 (4)	0.028 (4)	0.023 (4)	−0.006 (3)	−0.002 (3)	0.002 (3)
N3	0.022 (4)	0.030 (4)	0.027 (4)	0.000 (3)	−0.005 (3)	−0.005 (3)
N4	0.041 (5)	0.026 (4)	0.033 (5)	−0.005 (4)	−0.013 (4)	−0.008 (4)
N5	0.024 (4)	0.027 (4)	0.031 (4)	−0.004 (3)	−0.004 (3)	0.005 (3)
N6	0.027 (4)	0.043 (5)	0.023 (4)	−0.007 (4)	−0.006 (3)	0.006 (4)
C1	0.038 (6)	0.027 (5)	0.030 (5)	−0.008 (4)	−0.014 (4)	0.013 (4)
C2	0.033 (5)	0.026 (5)	0.043 (6)	0.001 (4)	−0.009 (5)	−0.005 (4)
C3	0.035 (6)	0.034 (6)	0.036 (6)	−0.011 (5)	−0.001 (4)	−0.009 (5)
C4	0.027 (5)	0.027 (5)	0.034 (5)	−0.012 (4)	−0.013 (4)	0.001 (4)
C5	0.036 (6)	0.042 (6)	0.024 (5)	−0.013 (5)	−0.013 (4)	0.000 (4)
C6	0.044 (7)	0.054 (7)	0.030 (6)	−0.013 (5)	−0.009 (5)	−0.015 (5)
C7	0.050 (7)	0.035 (6)	0.046 (7)	−0.016 (5)	−0.009 (5)	−0.008 (5)
C8	0.027 (5)	0.025 (5)	0.022 (5)	−0.004 (4)	−0.005 (4)	0.000 (4)
C9	0.031 (5)	0.042 (6)	0.037 (6)	−0.010 (5)	−0.019 (5)	0.002 (5)
C10	0.038 (6)	0.044 (6)	0.021 (5)	−0.013 (5)	−0.013 (4)	0.001 (4)
C11	0.040 (6)	0.050 (7)	0.016 (5)	−0.001 (5)	−0.009 (4)	−0.010 (4)
C12	0.042 (6)	0.025 (5)	0.016 (4)	−0.010 (4)	−0.009 (4)	0.009 (4)
O1	0.042 (4)	0.028 (4)	0.025 (4)	−0.010 (3)	−0.011 (3)	0.004 (3)

### Geometric parameters (Å, °)

**Table:**

I1—I2	2.9158 (10)	C2—C3	1.386 (15)
I1—I2^i^	2.9158 (10)	C2—H2	0.9500
I3—I4	2.9226 (11)	C3—H3	0.9500
I3—I4^ii^	2.9226 (11)	C4—C4^iii^	1.49 (2)
N1—C1	1.319 (13)	C5—C6	1.376 (15)
N1—C4	1.320 (12)	C5—H5	0.9500
N1—H1N	0.8800	C6—C7	1.363 (16)
N2—C4	1.339 (13)	C6—H6	0.9500
N2—C3	1.342 (13)	C7—H7	0.9500
N3—C5	1.324 (12)	C8—C8^iv^	1.525 (18)
N3—C8	1.342 (12)	C9—C10	1.370 (15)
N4—C8	1.316 (12)	C9—H9	0.9500
N4—C7	1.336 (14)	C10—C11	1.395 (15)
N5—C12	1.339 (13)	C10—H10	0.9500
N5—C9	1.355 (13)	C11—H11	0.9500
N6—C11	1.339 (13)	C12—C12^v^	1.484 (19)
N6—C12	1.348 (13)	O1—H1A	0.8400
C1—C2	1.385 (15)	O1—H1B	0.8400
C1—H1	0.9500		
			
I2—I1—I2^i^	180.00 (3)	C6—C5—H5	119.5
I4—I3—I4^ii^	180.0	C7—C6—C5	116.9 (10)
C1—N1—C4	117.8 (9)	C7—C6—H6	121.5
C1—N1—H1N	121.1	C5—C6—H6	121.5
C4—N1—H1N	121.1	N4—C7—C6	123.8 (10)
C4—N2—C3	116.1 (8)	N4—C7—H7	118.1
C5—N3—C8	117.3 (9)	C6—C7—H7	118.1
C8—N4—C7	114.8 (9)	N4—C8—N3	126.2 (9)
C12—N5—C9	117.8 (9)	N4—C8—C8^iv^	117.6 (10)
C11—N6—C12	115.7 (9)	N3—C8—C8^iv^	116.1 (10)
N1—C1—C2	121.9 (9)	N5—C9—C10	120.3 (9)
N1—C1—H1	119.0	N5—C9—H9	119.9
C2—C1—H1	119.0	C10—C9—H9	119.9
C1—C2—C3	116.5 (10)	C9—C10—C11	118.1 (9)
C1—C2—H2	121.8	C9—C10—H10	121.0
C3—C2—H2	121.8	C11—C10—H10	121.0
N2—C3—C2	122.0 (10)	N6—C11—C10	122.3 (9)
N2—C3—H3	119.0	N6—C11—H11	118.9
C2—C3—H3	119.0	C10—C11—H11	118.9
N1—C4—N2	125.6 (9)	N5—C12—N6	125.6 (9)
N1—C4—C4^iii^	117.7 (11)	N5—C12—C12^v^	117.6 (11)
N2—C4—C4^iii^	116.6 (10)	N6—C12—C12^v^	116.8 (11)
N3—C5—C6	120.9 (10)	H1A—O1—H1B	116.6
N3—C5—H5	119.5		
			
C4—N1—C1—C2	−1.3 (15)	C7—N4—C8—N3	−1.5 (15)
N1—C1—C2—C3	−0.8 (16)	C7—N4—C8—C8^iv^	−177.5 (11)
C4—N2—C3—C2	−0.8 (14)	C5—N3—C8—N4	2.7 (15)
C1—C2—C3—N2	1.8 (16)	C5—N3—C8—C8^iv^	178.8 (10)
C1—N1—C4—N2	2.5 (15)	C12—N5—C9—C10	−0.5 (14)
C1—N1—C4—C4^iii^	−179.0 (10)	N5—C9—C10—C11	−3.0 (15)
C3—N2—C4—N1	−1.5 (14)	C12—N6—C11—C10	−3.5 (15)
C3—N2—C4—C4^iii^	−179.9 (10)	C9—C10—C11—N6	5.2 (16)
C8—N3—C5—C6	−2.8 (15)	C9—N5—C12—N6	2.4 (14)
N3—C5—C6—C7	1.9 (16)	C9—N5—C12—C12^v^	−177.1 (10)
C8—N4—C7—C6	0.4 (16)	C11—N6—C12—N5	−0.4 (14)
C5—C6—C7—N4	−0.6 (18)	C11—N6—C12—C12^v^	179.1 (10)

Symmetry codes: (i) −*x*+2, −*y*, −*z*; (ii) −*x*, −*y*+1, −*z*+1; (iii) −*x*+1, −*y*, −*z*; (iv) −*x*+1, −*y*+1, −*z*+1; (v) −*x*, −*y*, −*z*+1.

### Hydrogen-bond geometry (Å, °)

**Table:**

*D*—H···*A*	*D*—H	H···*A*	*D*···*A*	*D*—H···*A*
O1—H1A···N3^iv^	0.84	1.88	2.721 (11)	178.
O1—H1B···N2^vi^	0.84	2.09	2.837 (10)	149.
N1—H1N···O1^vii^	0.88	2.59	3.450 (11)	166.

Symmetry codes: (iv) −*x*+1, −*y*+1, −*z*+1; (vi) −*x*+1, −*y*, −*z*+1; (vii) *x*, *y*, *z*−1.

## References

[bb1] Bruker (2000). *SADABS*, *SMART* and *SAINT* Bruker AXS Inc., Madison, Wisconsin, USA.

[bb3] Farrugia, L. J. (1997). *J. Appl. Cryst.* **30**, 565.

[bb2] Fialho De Assis, E., Howie, R. A. & Wardell, J. L. (1996). *Acta Cryst.* C**52**, 955–957.

[bb4] Ha, K. (2011). *Z. Kristallogr. New Cryst. Struct.* **226**, 365–367.

[bb5] Kochel, A. (2005). *Acta Cryst.* E**61**, m759–m760.

[bb6] Sheldrick, G. M. (2008). *Acta Cryst.* A**64**, 112–122. 10.1107/S0108767307043930 18156677

[bb7] Spek, A. L. (2009). *Acta Cryst.* D**65**, 148–155. 10.1107/S090744490804362X PMC2631630 19171970

